# Xenon Combined with Therapeutic Hypothermia Is Not Neuroprotective after Severe Hypoxia-Ischemia in Neonatal Rats

**DOI:** 10.1371/journal.pone.0156759

**Published:** 2016-06-02

**Authors:** Hemmen Sabir, Damjan Osredkar, Elke Maes, Thomas Wood, Marianne Thoresen

**Affiliations:** 1 Department of Physiology, Institute of Basic Medical Sciences, University of Oslo, Oslo, Norway; 2 Department of General Pediatrics, Neonatology and Pediatric Cardiology, Heinrich-Heine University, Duesseldorf, Germany; 3 School of Clinical Sciences, University of Bristol, St Michael’s Hospital, Bristol, United Kingdom; Robert Debre Hospital, FRANCE

## Abstract

**Background:**

Therapeutic hypothermia (TH) is standard treatment following perinatal asphyxia in newborn infants. Experimentally, TH is neuroprotective after moderate hypoxia-ischemia (HI) in seven-day-old (P7) rats. However, TH is not neuroprotective after severe HI. After a moderate HI insult in newborn brain injury models, the anesthetic gas xenon (Xe) doubles TH neuroprotection. The aim of this study was to examine whether combining Xe and TH is neuroprotective as applied in a P7 rat model of severe HI.

**Design/Methods:**

120 P7 rat pups underwent a severe HI insult; unilateral carotid artery ligation followed by hypoxia (8% O_2_ for 150min at experimental normothermia (NT-37: T_rectal_ 37°C). Surviving pups were randomised to immediate NT-37 for 5h (n = 36), immediate TH-32: T_rectal_ 32°C for 5h (n = 25) or immediate TH-32 plus 50% inhaled Xe for 5h (n = 24). Pups were sacrificed after one week of survival. Relative area loss of the ligated hemisphere was measured, and neurons in the subventricular zone of this injured hemisphere were counted, to quantify brain damage.

**Results:**

Following the HI insult, median (interquartile range, IQR) hemispheric brain area loss was similar in all groups: 63.5% (55.5–75.0) for NT-37 group, 65.0% (57.0–65.0) for TH-32 group, and 66.5% (59.0–72.0) for TH-32+Xe50% group (not significant). Correspondingly, there was no difference in neuronal cell count (NeuN marker) in the subventricular zone across the three treatment groups.

**Conclusions:**

Immediate therapeutic hypothermia with or without additional 50% inhaled Xe, does not provide neuroprotection one week after severe HI brain injury in the P7 neonatal rat. This model aims to mimic the clinical situation in severely asphyxiated neonates and treatment these newborns remains an ongoing challenge.

## Introduction

Therapeutic hypothermia (TH) reduces death and severe disability in newborn infants suffering moderate to severe perinatal asphyxia. However, 40–50% of these infants still have poor developmental outcome [1]. Therefore, improving outcome in asphyxiated newborns remains an ongoing challenge. Both pre-clinical animal model studies [2, 3] and randomised controlled trials [1] indicate, that TH best improves outcome in asphyxiated newborns with moderate brain injury applied within a limited time window of <6 hours after birth [4, 5]. Following severe asphyxial brain injury, however, TH seems to have a reduced effect, or even no effect [5]. This may explain the significant proportion of asphyxiated newborns that still have a poor outcome [1]. Early identification and treatment still comprises an important ongoing clinical challenge demanding the development of improved treatment options.

The anesthetic gas xenon (Xe) has been shown to double neuroprotection after moderate hypoxic-ischemic (HI) brain injury in newborn animal models when combined with TH [6–8]. When inhaled in a sub-anesthetic dosage of 50%, Xe is both neuroprotective and stabilizes the blood pressure and cerebrovascular reactivity [7, 9]. Due to the additive neuroprotective effect of TH+Xe after moderate experimental HI seen after both short (1 week) [10]and long term (adulthood) survival [6, 7, 11], TH and Xe is a promising combination treatment for infants with severe hypoxic brain injury. Approved safety studies in pigs [7, 12] led to the clinical feasibility study of adding Xe as treatment in infants undergoing TH after perinatal asphyxia [13]. This step-up dose study with 18 month follow-up showed that it was feasible to treat with 50% inhaled Xe for 18 hours while undergoing 72h of TH [13]. Recently, a randomised clinical trial combining treatment of 30% Xe for 24h with 72h TH in asphyxiated infants was published, showing no effect of the combined treatment after moderate or severe asphyxia [14]. A recent study in adults examined TH alone versus TH+50%Xe for 24h after out of hospital cardiac arrest [15]. They found less white matter injury on MRI scan examined 2–3 days after the arrest in the TH+50%Xe group, but no difference in death or neurological outcome after 6 months.

Experimentally, following severe HI, immediate TH is not neuroprotective after short-term survival in seven day old rats [5]. However, it is unknown, if immediate TH combined with 50% Xe will improve short and long term outcome following experimental severe HI.

## Material & Methods

### Procedures

All experiments were approved by the University of Bristol`s (30/2729) and University of Oslo’s (12–4343) animal ethics research committees. Experiments were performed on 7-day-old (P7) Wistar rats of both sexes randomised prior to treatment across litter, sex and weight. Dams with pups were kept in an animal facility with a 12:12h dark:light cycle at 21°C with food and water ad libitum. Pups were weighed and checked for health daily.

### Severe Hypoxic-Ischemic Injury

One-hundred and twenty P7 rat pups of both sexes from 12 litters underwent a left common carotid ligation under general anesthesia, followed by recovery with their dam for a minimum of 30min, as previously described [5]. After a maximum delay of 180min from the time of ligation, pups were exposed to 8% oxygen for 150min at a rectal temperature (T_rectal_) of 37.0°C in a temperature-controlled chamber. This results in a severe hypoxic-ischemic insult with around 60% hemispheric area loss [5]. A T_rectal_ of 37.0°C±0.2°C was achieved within 15min. Rectal temperature correlates within 0.1°C of brain temperature in P7 rats [16]. The temperature was continuously measured in “sentinel” pups with a rectal temperature probe (IT-21, Physitemp Instruments, Clifton, USA, n = 6) or a skin probe (CritiCool, MTRE, Yavne, Israel, n = 6) on the abdomen and maintained with a servo-controlled mat (CritiCool, MTRE, Yavne, Israel). Each treatment group included two sentinel pups at the same time in each treatment chamber (one rectal probe, one skin probe). The water temperature in the mat, on which the pups were resting, was servo-controlled by the rectal probe animal. Both probes were calibrated to ±0.1°C over a range of 20.0 to 40.0°C against a certified mercury-in-glass thermometer (BS593; Zeal, London, UK). Twenty-three pups died during this severe hypoxic insult. Animals that carried a temperature probe were excluded from further analysis (n = 12). After excluding deaths and probe animals, eighty-five pups were allocated (Table 1) to one of the following treatments: immediate experimental normothermia at T_rectal_ 37°C for 5h in air (NT-37, n = 36); immediate hypothermia at T_rectal_ 32°C for 5h in air (TH-32, n = 25); or immediate TH-32 plus 50% inhaled Xe for 5h (TH-32 + Xe50%, n = 24). In the treatment groups, both a T_rectal_ of 32.0°C±0.2°C and a stable Xe concentration was achieved within 15min. In the Xe group, the Xe concentration within the chamber was monitored continuously using our previously described closed re-circulating system that conserves Xe [6]. During the treatment period the condition of the pups was visually monitored within the treatment chamber every five minutes. We have previously shown that P7 pups have normal respiratory drive and pCO_2_ values whilst being cooled with or without 50% Xe delivery [17]. No clinical seizures were observed. After the treatment period, pups were immediately removed from the chamber and returned to their dam.

**Table 1:** Weight at 7 days of age (P7) and weight gain after 7 days of survival (P14) across the three treatment groups. Values are median (IQR). Treatment groups are NT-37: normothermia, kept at T_rectal_ 37°C for 5h; TH-32: hypothermia, kept at T_rectal_ 32°C for 5h; TH-32+Xe50%: combined treatment of TH-32 and 50% inhaled Xe for 5h.

	NT-37	TH-32	TH-32 + Xe 50%
**Total number, Sex**	n = 36, 18 Male	n = 25, 12 Male	n = 24, 13 Male
**Weight P7**	12g (12.0–13.0)	12g (11.0–13.5)	12.5g (11.0–13.0)
**Weight P14**	19.5g (18.0–21.0)	20g (18.0–21.5)	20g (18.3–22.8)

### Area Measurement (Brain Area Loss)

**Fig 1:**
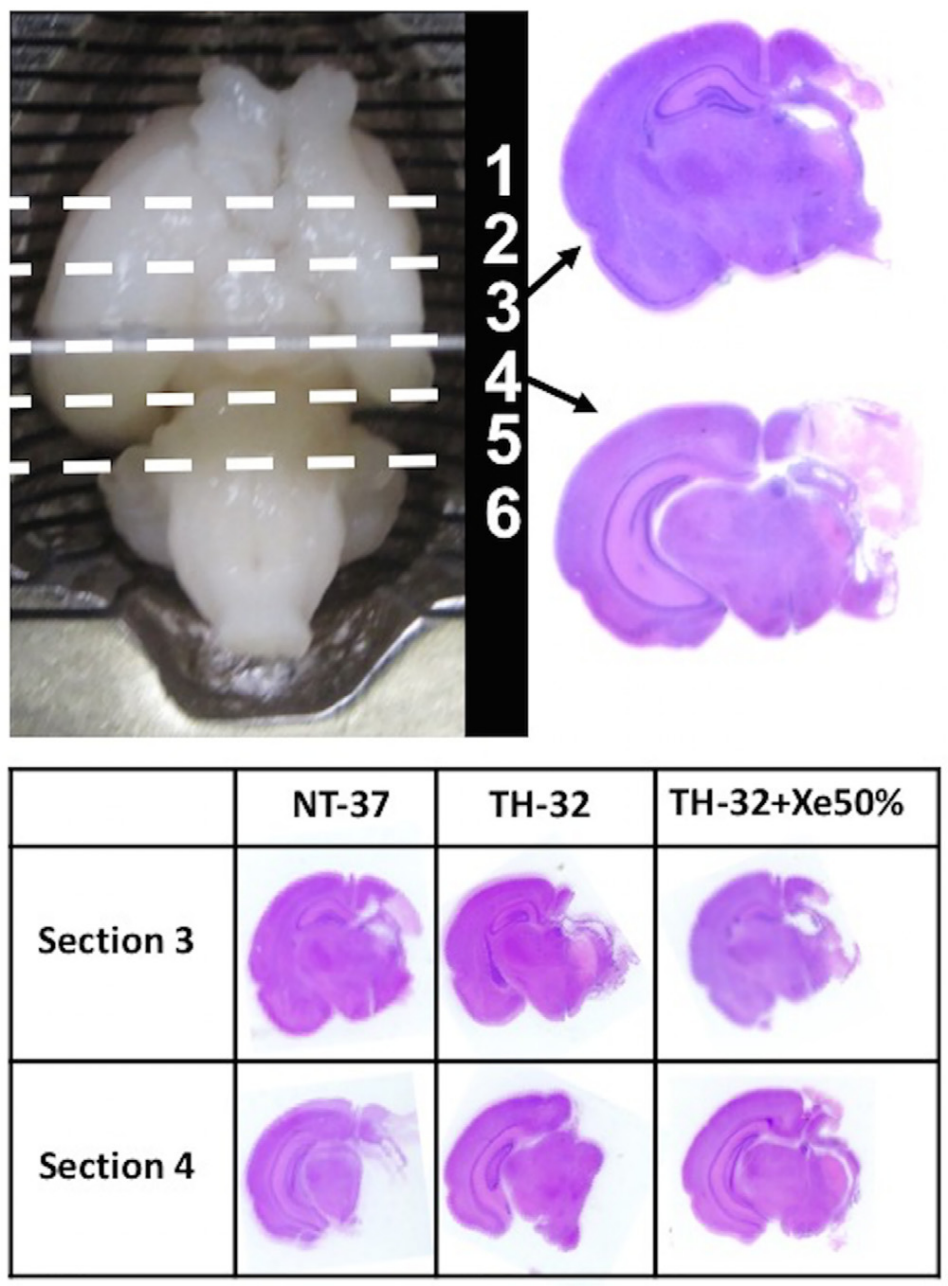
Rat brain placed in a standard matrix for uniformity. The rat brain is cut in 6 coronal blocks. Of these, block number 3 and 4 were used for further processing and analysis and stained with hematoxylin & eosin. Representing sections of animals from the different treatment groups, showing severely damaged brains in both sections and in every treatment group.

After seven days of survival all animals were deeply anesthetised with isoflurane/N_2_O. After sufficient anesthesia transcardiac perfusion with 10% neutral-buffered formalin was performed before decapitation. Brains were manually removed and kept in 10% neutral-buffered formalin until further processing [5, 10]. Coronal 3mm blocks were cut through the brain using a standard matrix for uniformity (ASI Instruments Inc., Warren, USA) and were embedded in paraffin. Blocks were further cut into 5 μm sections and stained with haematoxylin and eosin (H&E) (Fig 1). Two sections from each of two neighbouring blocks representing cortex, hippocampus, basal ganglia and thalamus, were scanned (Epson, Perfection V30, Telford, UK) at 1200 dpi resolution. To measure the area of brain tissue loss, the image of each section was opened in ImageJ software (ImageJ, version 1.43, National Institutes of Health, USA), by an individual blinded to the experimental allocation. The midline of each brain section was identified on the image and the brain divided by its hemispheres (left vs right), and the viable tissue in the left and right hemisphere measured, as previously described [5, 10]. The ratio of the hemispheric areas was calculated for the two sections per brain and the average percentage of area loss was calculated respectively ((1—(Area Ratio (left vs right)) x 100).

### Immunohistochemistry and Neuronal Cell Counting in the Subventricular Zone

To evaluate a more detailed effect of different treatments on neuronal injury in a representative subset of animals, immunohistochemistry analysis was performed at P14 using NeuN, a neuronal marker, as previously described [18]. Paraffin-embedded tissue was deparaffinised in xylene and rehydrated in decreasing concentrations of ethanol. Antigen retrieval was then performed in a citrate buffer solution pH 6.0, using a PT link instrument (Dako, Glostrup, Denmark). After blocking in 10% goat serum, primary rabbit antibody against NeuN (1:500; Millipore) was applied overnight at room temperature. After rinsing with PBS, the slices were incubated for 1 h at room temperature with secondary Alexa Fluor 568 and/or 488 (Invitrogen, 1:500) antibodies. Finally, the slides were rinsed and coverslipped with ProLong Gold with DAPI (Invitrogen). Sections were scanned with a virtual microscopy scanner (Axio Scan.Z1; Carl Zeiss, Jena, Germany) using the fluorescence mode with plan apochromatic 20× lens. Virtual slides were exported as high-resolution tiff images for further analysis.

**Fig 2:**
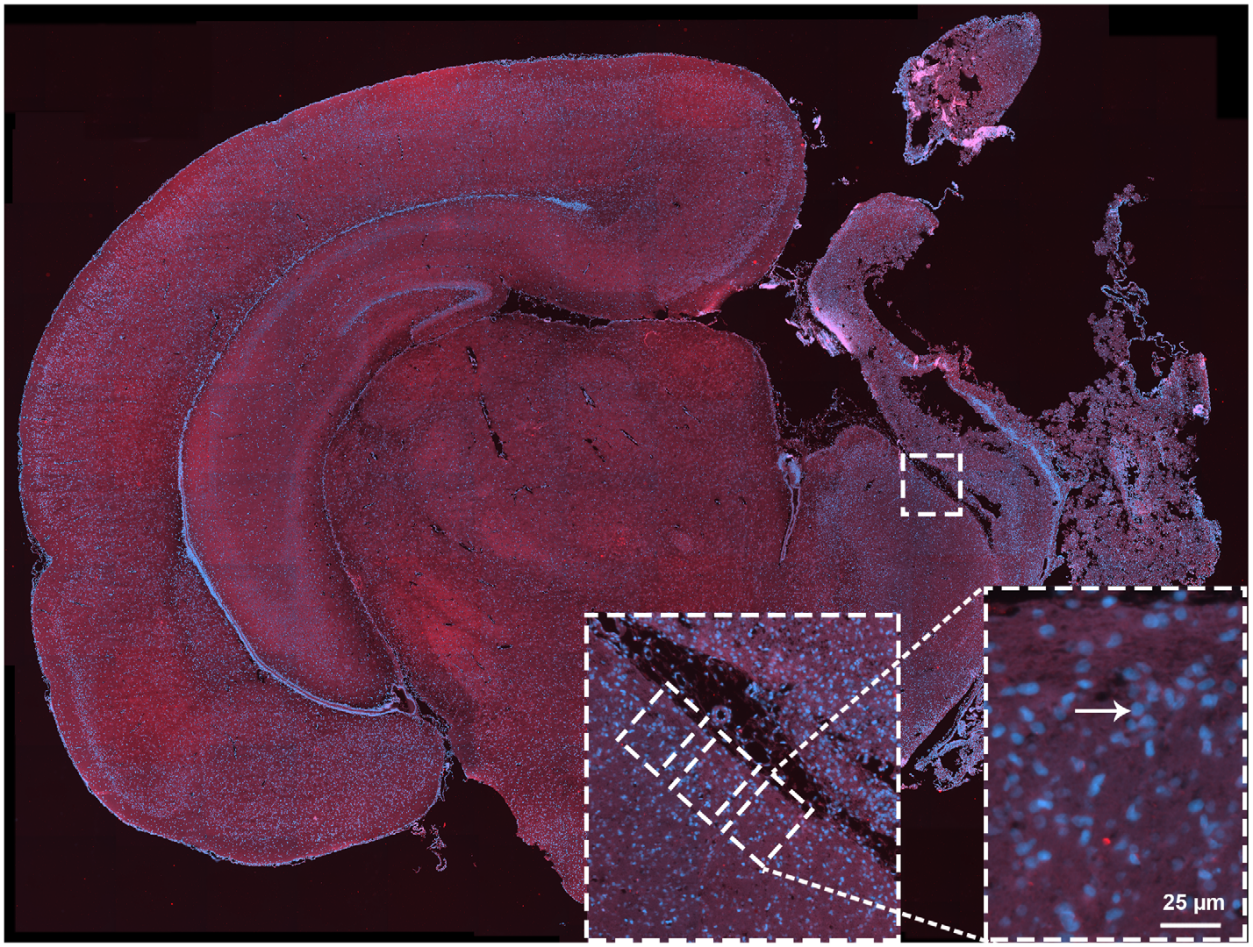
Scanned mid-brain section best representing the cortex, hippocampus, basal ganglia and thalamus. Neuronal cell counting was performed in the subventricular zone. A customised grid was used to count cells in three non-overlapping fields. Each analysed brain had the same sized grid applied at the mid-ventricular region as depicted by the white rectangle. Neurons were counted if they displayed large, round nuclei (DAPI, blue) and NeuN co-staining (red).

To quantify neuronal injury, we have previously analysed the area of the hippocampus, as main region of interest after mild and moderate HI [19]. However, due to the extent of hippocampal damage and tissue loss in the severe model, the hippocampus could not be analysed [5]. Therefore, to quantify the effect of different treatments on neuronal injury, neurons in the subventricular zone were counted, as this region is also highly vulnerable after HI in newborn rats (Fig 2) [20]. Viable neurons from three non-overlapping fields from 13 animals per group were counted by an individual blinded to the treatment groups, as previously described [18]. A standardised grid was created and applied to the mid-ventricular section of each image using virtual microscopy scanner software (Axio Scan.Z1; Carl Zeiss, Jena, Germany).

### Data Analysis

Statistical analyses were performed with SPSS version 22 (SPSS Inc., Chicago, USA). For two-group comparisons the Wilcoxon-Mann-Whitney U-test was used. One way ANOVA was used to compare the different treatment groups. Effects of sex and weight of pups on brain area loss were analysed by linear regression. Two-sided testing with *p*<0.05 was considered statistically significant. Descriptive data are presented as median and interquartile range (IQR).

## Results

There was no significant difference between the groups regarding sex, weight at P7 or weight gain at P14 (Table 1). Twenty-one percent died during the severe HI insult.

### Brain Area Loss

**Fig 3:**
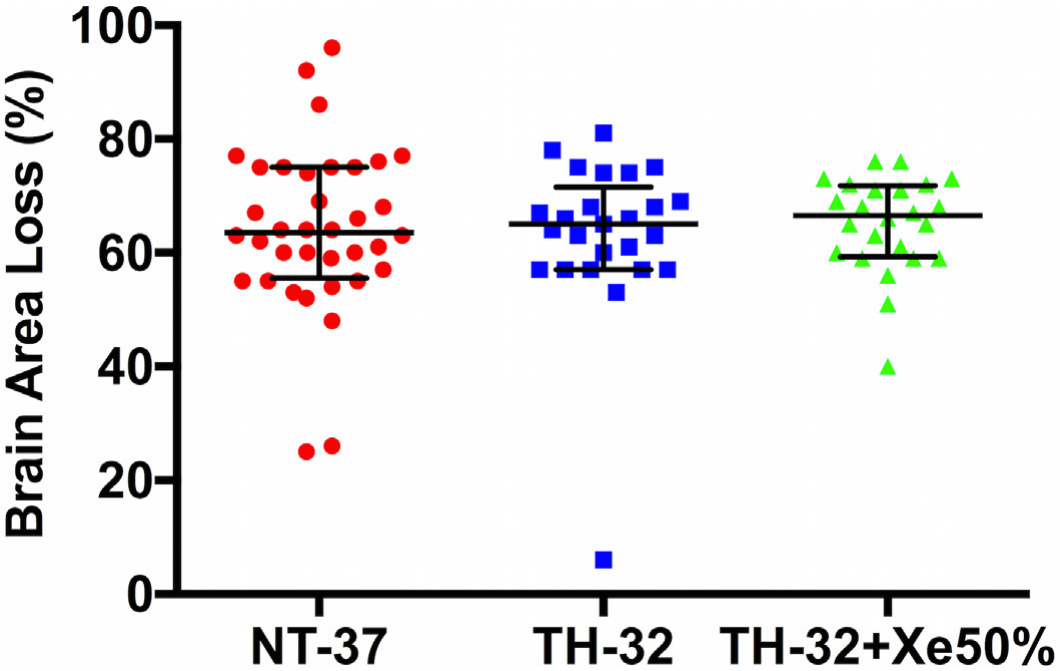
Vertical scatter plot with median (IQR) brain area loss across the different treatment groups. There was no reduction in brain area loss in either the therapeutic hypothermia group or in the combined therapeutic hypothermia plus 50% inhaled xenon group. NT-37: normothermia at T_rectal_ 37°C for 5h (n = 36); TH-32: hypothermia at T_rectal_ 32°C for 5h (n = 25); TH-32 + Xe50%: TH-32 plus 50% inhaled Xenon for 5h (n = 24).

The median (IQR) brain area loss was 63.5% (55.5–75.0) in the NT-37 group, 65.0% (57.0–65.0) in the TH-32 group, and 66.5% (59.0–72.0) in the TH-32 + Xe50% group (Fig 3). Linear regression showed no significant effect of sex or weight on brain area loss.

### Neuronal Cell Counting in Subventricular Zone

**Fig 4:**
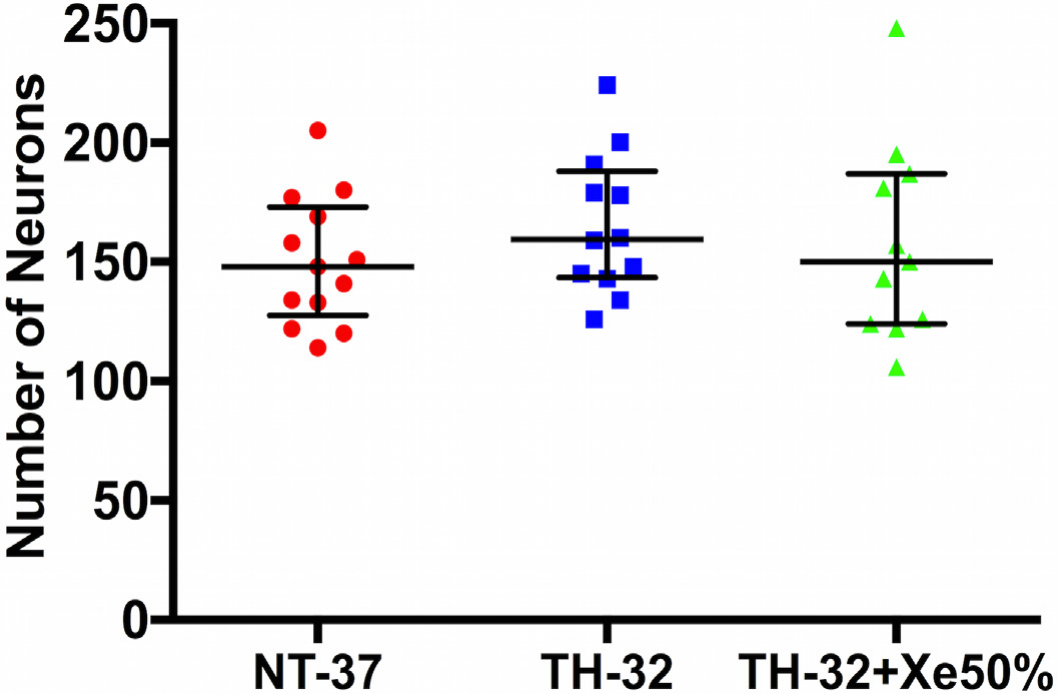
Vertical scatter plot with median (IQR) number of neurons in the subventricular zone. There was no significant difference between the different treatment groups (n = 13 per group).

There was no difference in the number of NeuN stained neurons in the subventricular zone between the different treatment groups (Fig 4). Median (IQR) number of counted neurons for the different treatment groups were 148.0 (127.5–173.0) in the NT-37 group; 159.5 (143.5–188.0) in the TH-32 group; 150.0 (124.0–187.0) in the TH-32 + Xe50 group.

## Discussion

This study shows that neither immediate hypothermia alone, nor combined with 50% inhaled Xenon, provides short term neuroprotection after severe hypoxia-ischemia in an established neonatal rat model of severe hypoxic-ischemic (HI) brain injury. This is consistent with our previous findings [5]. We have previously developed this severe HI injury neonatal rat model by increasing the duration of hypoxia from 90 to 150min and temperature during the hypoxic-ischemic insult from 36°C to 37°C, compared to the standard Vannucci moderate brain injury model [5].

It is important to establish early safe treatment options, combined with TH, to improve outcome in severely asphyxiated newborns. Early biochemical or physiological markers that can be used to identify the infants at the highest-risk are required, however this small animal model with injury to the brain, but not the body, is not suitable for this global question.

Xenon, an inhalational anesthetic, has been shown to additively double neuroprotection in both this neonatal Vannucci rat brain injury model, as well as in the global hypoxia-ischemia newborn pig model [6, 7, 10]. This is thought to be mainly due to antagonism of the N-methyl-d-aspartate (NMDA) glutamatergic receptor [21]. Xenon is a low-affinity non-competitive NMDA receptor antagonists that reduces glutamate-triggered excitotoxicity [22]. Importantly, in contrast to high-affinity NMDA receptor antagonists, Xe does not appear to have any neurotoxic effects [23]. When administered at a sub-anesthetic dosage (50%), Xe also stabilises blood pressure and cardiac output and reduces the need for inotropes [9]. This might be partly due to increased neuronal norepinephrine levels as a result of Xe-mediated antagonism of norepinephrine reuptake [24].

We have shown previously, that breathing 50% Xe for 24h is safe and does not increase neuroapoptosis in healthy newborn pigs when combined with additional opioid sedation as used clinically [25]. As Xe with TH is additively neuroprotective in preclinical models of moderate HI [6, 7, 10], we hypothesised that adding 50% Xe to TH might be a beneficial treatment option following severe HI. There are some limitations to our study. Firstly, we have not undertaken a long term survival study in the severe model and may therefore have missed a long term positive effect of Xe. As shown in this animal model, certain short-term neuropathological markers do not always predict long-term injury [26] and neurobehavioural long term improvement can be seen that are not predicted solely by short-term pathology [27]. In a recent long term study on delayed TH and Xe with survival to adulthood, we found that the functional recovery had normalised on testing, and was significantly better in the Xe+TH group as compared to TH alone [11], despite a non-significant difference in hemispheric area loss. Recently, a short term clinical survival study did not find an additional neuroprotective effect of adding 30% Xe to TH in asphyxiated newborns [14]. Xenon was added late, at a median age of 10h after birth. However, with these limitations in design and follow up, there is still a possibility that adding Xe to TH is neuroprotective and that we have not yet developed the optimal protocol. Since no neuroprotective effect was noted after the combined treatment with TH and Xe in the severe model, it is possible that the Xe concentration, duration or survival time was too short to find an effect. For instance, Robertson et al. have shown that 30% Xe administered for 24h in combination with TH resulted in additional neuroprotection in a newborn pig model of moderate encephalopathy [12]. Franks et al. and Ma et al. have both shown a dose-response relationship between the dose of Xe and its neuroprotective potency [8, 26]. We have previously shown in newborn rats and pigs, that 50% inhaled Xe combined with TH doubles neuroprotection, and therefore chosen this concentration in the current study [6, 7, 10]. Fifty percent inhaled Xe is a feasible concentration to be delivered to asphyxiated newborns, as most asphyxiated newborns require some oxygen treatment [13]. Additionally, changing the temperature following severe HI injury might be an additional treatment option. However, it has been shown by Wood et al. that neither increasing nor lowering the temperature within the range 26–33.5°C following severe HI improves neuroprotection in this animal model [18]. Instead, lower temperatures (<26°C) resulted in an increase of injury. Secondly, during NT, 37°C was maintained as normothermia for P7 rats. 37°C has historically been used by us and many other groups being the NT control temperature to compare with a period of TH treatments, as it was assumed to be the normal core temperature in P7 rats [2, 25]. However, normothermia may vary widely depending on local experimental conditions and it is likely that 37°C is relatively hyperthermic for a P7 newborn rat [18]. Thirdly, the maturation of a 7 day old rat brain, as originally described by Rice and Vannucci [27], corresponds to a preterm human of around 32–36 weeks of gestation [28, 29]. A P10 rat is thought to be closer to a term human infant with regards to brain maturation. However the P7 model has been intensively studied by many groups worldwide, is thought to sufficiently model the term, and has been used to obtain seminal information about the pathophysiology of HI brain injury [30]. Finally, we did not investigate whether 50% Xe alone is neuroprotective in this study. However, we have previously shown that the combined treatment effect of TH plus Xe is based on additional neuroprotection and not synergism [6, 10]. Therefore, we would not expect any significant neuroprotective effect in Xe only treatment group.

Currently, inhaled Xe, in addition to TH, for the treatment of neonatal encephalopathy of hypoxic-ischemic origin has been implemented in two clinical trials, one of which (TOBYXe) was recently published [14]. Azzopardi et al. have combined 30% Xe for 24h started within the first 12h after birth with standard cooling started within the first 6h after birth. The authors found no significant differences in short term outcome markers between the two treatment groups and the study stopped recruiting early. There are a number of potential reasons for the lack of efficiency in this trial. Firstly, the onset of Xe ventilation was likely to be outside the therapeutic time window of additional neuroprotection. Secondly, even though there was no significant difference in the HIE severity scores between the two treatment groups, the newborns in the cooling plus Xe group had slightly higher encephalopathy scores at trial entry and during the first week of life. Xe might also exert a positive developmental effect longer term, and an MR examination by 1 week is too early.

Another ongoing clinical trial with both early MR/MRS outcomes and 18 months follow up, CoolXenon (ClinicalTrials.gov Identifier: NCT01545271), is still recruiting patients and combines 50% Xe for 18h (started within the first 5h of birth) with 72h cooling to 33.5°C (started within 3h of birth). The two different treatment protocols in the different trials (TobyXe versus CoolXenon) will answer important questions regarding timing and concentration of Xe administration.

Experimentally, immediate therapeutic hypothermia with or without additional 50% inhaled Xe, does not appear to provide short term neuroprotection after severe HI brain injury in neonatal P7 rats.

## References

[1] Jacobs SE, Berg M, Hunt R, Tarnow-Mordi WO, Inder TE, Davis PG. Cooling for newborns with hypoxic ischaemic encephalopathy. The Cochrane database of systematic reviews. 2013;1:Cd003311 Epub 2013/02/27. 10.1002/14651858.CD003311.pub3 . 23440789 PMC7003568

[2] Bona E, Hagberg H, Loberg EM, Bagenholm R, Thoresen M. Protective effects of moderate hypothermia after neonatal hypoxia-ischemia: short- and long-term outcome. Pediatric research. 1998;43(6):738–45. Epub 1998/06/11. 10.1203/00006450-199806000-00005 . 9621982

[3] Tooley JR, Satas S, Porter H, Silver IA, Thoresen M. Head cooling with mild systemic hypothermia in anesthetized piglets is neuroprotective. Annals of neurology. 2003;53(1):65–72. Epub 2003/01/02. 10.1002/ana.10402 . 12509849

[4] Gunn AJ, Bennet L, Gunning MI, Gluckman PD, Gunn TR. Cerebral hypothermia is not neuroprotective when started after postischemic seizures in fetal sheep. Pediatric research. 1999;46(3):274–80. Epub 1999/09/03. 10.1203/00006450-199909000-00005 . 10473041

[5] Sabir H, Scull-Brown E, Liu X, Thoresen M. Immediate hypothermia is not neuroprotective after severe hypoxia-ischemia and is deleterious when delayed by 12 hours in neonatal rats. Stroke; a journal of cerebral circulation. 2012;43(12):3364–70. Epub 2012/09/22. 10.1161/strokeaha.112.674481 . 22996953

[6] Hobbs C, Thoresen M, Tucker A, Aquilina K, Chakkarapani E, Dingley J. Xenon and hypothermia combine additively, offering long-term functional and histopathologic neuroprotection after neonatal hypoxia/ischemia. Stroke; a journal of cerebral circulation. 2008;39(4):1307–13. Epub 2008/03/01. 10.1161/strokeaha.107.499822 . 18309163

[7] Chakkarapani E, Dingley J, Liu X, Hoque N, Aquilina K, Porter H, et al Xenon enhances hypothermic neuroprotection in asphyxiated newborn pigs. Annals of neurology. 2010;68(3):330–41. Epub 2010/07/27. 10.1002/ana.22016 . 20658563

[8] Ma D, Hossain M, Chow A, Arshad M, Battson RM, Sanders RD, et al Xenon and hypothermia combine to provide neuroprotection from neonatal asphyxia. Annals of neurology. 2005;58(2):182–93. Epub 2005/07/29. 10.1002/ana.20547 . 16049939

[9] Chakkarapani E, Dingley J, Aquilina K, Osredkar D, Liu X, Thoresen M. Effects of xenon and hypothermia on cerebrovascular pressure reactivity in newborn global hypoxic-ischemic pig model. Journal of cerebral blood flow and metabolism: official journal of the International Society of Cerebral Blood Flow and Metabolism. 2013;33(11):1752–60. Epub 2013/08/01. 10.1038/jcbfm.2013.123 ; PubMed Central PMCID: PMCPmc3824173. 23899927 PMC3824173

[10] Sabir H, Walloe L, Dingley J, Smit E, Liu X, Thoresen M. Combined treatment of xenon and hypothermia in newborn rats—additive or synergistic effect? PloS one. 2014;9(10):e109845 Epub 2014/10/07. 10.1371/journal.pone.0109845 ; PubMed Central PMCID: PMCPmc4186877. 25286345 PMC4186877

[11] Liu X, Dingley J, Scull-Brown E, Thoresen M. Adding 5 h delayed xenon to delayed hypothermia treatment improves long-term function in neonatal rats surviving to adulthood. Pediatric research. 2015;77(6):779–83. Epub 2015/03/12. 10.1038/pr.2015.49 . 25760545

[12] Faulkner S, Bainbridge A, Kato T, Chandrasekaran M, Kapetanakis AB, Hristova M, et al Xenon augmented hypothermia reduces early lactate/N-acetylaspartate and cell death in perinatal asphyxia. Annals of neurology. 2011;70(1):133–50. Epub 2011/06/16. 10.1002/ana.22387 . 21674582

[13] Dingley J, Tooley J, Liu X, Scull-Brown E, Elstad M, Chakkarapani E, et al Xenon ventilation during therapeutic hypothermia in neonatal encephalopathy: a feasibility study. Pediatrics. 2014;133(5):809–18. Epub 2014/04/30. 10.1542/peds.2013-0787 . 24777219

[14] Azzopardi D, Robertson NJ, Bainbridge A, Cady E, Charles-Edwards G, Deierl A, et al Moderate hypothermia within 6 h of birth plus inhaled xenon versus moderate hypothermia alone after birth asphyxia (TOBY-Xe): a proof-of-concept, open-label, randomised controlled trial. The Lancet Neurology. 2015 Epub 2015/12/29. 10.1016/s1474-4422(15)00347-6 . 26708675 PMC4710577

[15] Laitio R, Hynninen M, Arola O, Virtanen S, Parkkola R, Saunavaara J, et al Effect of Inhaled Xenon on Cerebral White Matter Damage in Comatose Survivors of Out-of-Hospital Cardiac Arrest: A Randomized Clinical Trial. Jama. 2016;315(11):1120–8. Epub 2016/03/16. 10.1001/jama.2016.1933 . 26978207

[16] Thoresen M, Bagenholm R, Loberg EM, Apricena F, Kjellmer I. Posthypoxic cooling of neonatal rats provides protection against brain injury. Archives of disease in childhood Fetal and neonatal edition. 1996;74(1):F3–9. Epub 1996/01/01. ; PubMed Central PMCID: PMCPmc2528334. 8653432 10.1136/fn.74.1.f3 PMC2528334

[17] Dingley J, Tooley J, Porter H, Thoresen M. Xenon provides short-term neuroprotection in neonatal rats when administered after hypoxia-ischemia. Stroke; a journal of cerebral circulation. 2006;37(2):501–6. Epub 2005/12/24. 10.1161/01.STR.0000198867.31134.ac . 16373643

[18] Wood T, Osredkar D, Puchades M, Maes E, Falck M, Flatebo T, et al Treatment temperature and insult severity influence the neuroprotective effects of therapeutic hypothermia. Sci Rep. 2016;6:23430 Epub 2016/03/22. 10.1038/srep23430 26997257 PMC4800445

[19] Osredkar D, Thoresen M, Maes E, Flatebo T, Elstad M, Sabir H. Hypothermia is not neuroprotective after infection-sensitized neonatal hypoxic-ischemic brain injury. Resuscitation. 2014;85(4):567–72. Epub 2013/12/24. 10.1016/j.resuscitation.2013.12.006 . 24361672

[20] Romanko MJ, Zhu C, Bahr BA, Blomgren K, Levison SW. Death effector activation in the subventricular zone subsequent to perinatal hypoxia/ischemia. Journal of neurochemistry. 2007;103(3):1121–31. Epub 2007/08/23. 10.1111/j.1471-4159.2007.04820.x . 17711427

[21] Dickinson R, Peterson BK, Banks P, Simillis C, Martin JC, Valenzuela CA, et al Competitive inhibition at the glycine site of the N-methyl-D-aspartate receptor by the anesthetics xenon and isoflurane: evidence from molecular modeling and electrophysiology. Anesthesiology. 2007;107(5):756–67. Epub 2007/12/13. 10.1097/01.anes.0000287061.77674.71 . 18073551

[22] David HN, Haelewyn B, Rouillon C, Lecoq M, Chazalviel L, Apiou G, et al Neuroprotective effects of xenon: a therapeutic window of opportunity in rats subjected to transient cerebral ischemia. FASEB journal: official publication of the Federation of American Societies for Experimental Biology. 2008;22(4):1275–86. Epub 2007/11/21. 10.1096/fj.07-9420com . 18024836

[23] Palmer GC, Widzowski D. Low affinity use-dependent NMDA receptor antagonists show promise for clinical development. Amino acids. 2000;19(1):151–5. Epub 2000/10/12. . 11026483 10.1007/s007260070043

[24] Neukirchen M, Hipp J, Schaefer MS, Brandenburger T, Bauer I, Winterhalter M, et al Cardiovascular stability and unchanged muscle sympathetic activity during xenon anaesthesia: role of norepinephrine uptake inhibition. British journal of anaesthesia. 2012;109(6):887–96. Epub 2012/09/05. 10.1093/bja/aes303 . 22945969

[25] Sabir H, Bishop S, Cohen N, Maes E, Liu X, Dingley J, et al Neither xenon nor fentanyl induces neuroapoptosis in the newborn pig brain. Anesthesiology. 2013;119(2):345–57. Epub 2013/04/18. 10.1097/ALN.0b013e318294934d . 23591070

[26] Franks NP, Dickinson R, de Sousa SL, Hall AC, Lieb WR. How does xenon produce anaesthesia? Nature. 1998;396(6709):324 Epub 1998/12/09. 10.1038/24525 . 9845069

[27] Rice JE 3rd, Vannucci RC, Brierley JB. The influence of immaturity on hypoxic-ischemic brain damage in the rat. Annals of neurology. 1981;9(2):131–41. Epub 1981/02/01. 10.1002/ana.410090206 . 7235629

[28] Hagberg H, Peebles D, Mallard C. Models of white matter injury: comparison of infectious, hypoxic-ischemic, and excitotoxic insults. Mental retardation and developmental disabilities research reviews. 2002;8(1):30–8. Epub 2002/03/29. 10.1002/mrdd.10007 . 11921384

[29] Semple BD, Blomgren K, Gimlin K, Ferriero DM, Noble-Haeusslein LJ. Brain development in rodents and humans: Identifying benchmarks of maturation and vulnerability to injury across species. Progress in neurobiology. 2013;106–107:1–16. Epub 2013/04/16. 10.1016/j.pneurobio.2013.04.001 23583307 PMC3737272

[30] Mallard C, Vexler ZS. Modeling Ischemia in the Immature Brain: How Translational Are Animal Models? Stroke; a journal of cerebral circulation. 2015;46(10):3006–11. Epub 2015/08/15. 10.1161/strokeaha.115.007776 26272384 PMC4589478

